# Post-operative Outcomes of Laparoscopic Versus Open Repair of Perforated Duodenal Ulcer: A Systematic Review

**DOI:** 10.7759/cureus.92021

**Published:** 2025-09-10

**Authors:** Parin Y Patel, Milind K Akhani, Ronak Rathod, Bhavin Baria

**Affiliations:** 1 General Surgery, Health1 Super Speciality Hospitals, Ahmedabad, IND; 2 Gastroenterology, Health1 Super Speciality Hospitals, Ahmedabad, IND; 3 General Surgery, Shalby Multi-Specialty Hospitals, Ahmedabad, IND

**Keywords:** comparative study, duodenal ulcer perforation, hospital length of stay, laparoscopic repair, mortality rate, open repair

## Abstract

Perforated duodenal ulcer is a surgical emergency associated with substantial morbidity and mortality. While open repair has long been the standard, laparoscopic repair is increasingly adopted for its minimally invasive advantages. This systematic review, conducted in accordance with Preferred Reporting Items for Systematic Reviews and Meta-Analyses (PRISMA) 2020 guidelines and registered with PROSPERO (CRD420251117651), compared outcomes of laparoscopic versus open repair in adult patients with perforated duodenal ulcers. A comprehensive search of PubMed, Embase, and the Cochrane Library identified comparative studies published between January 2000 and December 2024. Six studies involving 739 patients met the inclusion criteria. Outcomes assessed included operative time, conversion rate, length of hospital stay (LOS), post-operative complications, leak rate, and mortality. Risk of bias was evaluated using the Cochrane Risk of Bias 2 (RoB-2) tool for the single randomized controlled trial and the Newcastle-Ottawa Scale for observational studies.

Laparoscopic repair was consistently associated with shorter LOS (4.0-7.8 versus 7.8-11.7 days), lower complication rates (5.8-13% versus 8.6-44.3%), and reduced mortality (0-1.8% versus 0-27.9%) compared with open repair, while leak rates were comparable (0-7% versus 1.4-4.9%). Conversion to open surgery occurred in 0-17.8% of laparoscopic cases. Operative times were longer in earlier studies but equivalent or shorter in more recent work. Risk of bias ranged from low to moderate. In conclusion, laparoscopic repair is a safe and effective alternative to open repair, offering a faster recovery and lower morbidity without increasing the risk of leaks. Open repair remains essential for unstable patients or those with severe contamination. Further multicentre randomized trials with standardized outcome reporting are warranted to confirm these benefits and define optimal patient selection.

## Introduction and background

Peptic ulcer disease (PUD) remains a significant gastrointestinal condition worldwide. Although its incidence has declined in recent decades due to the widespread use of proton pump inhibitors and eradication therapies for *Helicobacter pylori*, complications, such as perforated duodenal ulcers, continue to carry considerable morbidity and mortality, particularly in emergency surgical settings and resource-limited environments. Prompt surgical management remains the cornerstone of treatment to reduce the risk of peritonitis and sepsis [1].

The open omental patch repair, first described by Graham in 1937, has long been established as the gold standard for surgical management of perforated duodenal ulcers [2]. This technique has proven to be reliable, reproducible, and widely applicable across varying levels of surgical infrastructure.

In recent decades, however, laparoscopic repair has gained increasing acceptance as a minimally invasive alternative to open surgery. Several studies have reported that laparoscopic repair may offer benefits including reduced post-operative pain, lower wound infection rates, shorter hospital stays, and faster return to normal activity, without compromising surgical safety or efficacy [3]. Despite these advantages, concerns remain regarding the technical difficulty, longer operative time, and the required surgeon experience associated with laparoscopic approaches, particularly in emergency scenarios [4].

While existing literature has evaluated laparoscopic and open techniques in the context of peptic ulcer perforation, many studies do not differentiate between gastric and duodenal perforations, which are anatomically and clinically distinct. Duodenal ulcers typically occur in younger patients, more often in the anterior wall, and may differ in perforation size, intra-abdominal contamination, and tissue fragility-factors that influence operative decision-making [5].

Given the need for a focused evaluation, the present review was therefore undertaken to systematically compare laparoscopic and open repair in the specific context of perforated duodenal ulcers. The primary aim was to evaluate differences in morbidity and mortality between the two approaches. Secondary aims included assessing operative time, conversion rates, length of hospital stay, surgical complications, such as wound and respiratory infections, and anastomotic leak rates.

## Review

Methodology

This systematic review was conducted in accordance with the Preferred Reporting Items for Systematic Reviews and Meta-Analyses (PRISMA) 2020 guidelines and was prospectively registered with the International Prospective Register of Systematic Reviews (PROSPERO) (CRD420251117651) [6].

Eligibility Criteria

Studies were included if they compared laparoscopic and open repair in adult patients (≥18 years) presenting with a perforated duodenal ulcer. Eligible designs included randomized controlled trials, prospective comparative studies, and retrospective comparative studies. Exclusion criteria were as follows: studies limited to gastric ulcer perforations without separate subgroup analysis, non-comparative case series, case reports, review articles, animal studies, and pediatric cohorts. Only studies published in English were included. Grey literature, such as conference abstracts and dissertations, was excluded due to incomplete methodological and outcome reporting. The eligibility criteria were structured according to the Population, Intervention, Comparator, Outcomes, and Study Design (PICOS) framework (Table 1).

**Table 1:** PICOS framework for eligibility criteria. PICOS: Population, Intervention, Comparator, Outcomes, and Study Design

Elements	Description
Population (P)	Adult patients (≥18 years) presenting with perforated duodenal ulcer
Intervention (I)	Laparoscopic omental patch repair
Comparator (C)	Open omental patch repair
Outcomes (O)	Primary: post-operative morbidity and mortality. Secondary: operative time, conversion rate, length of hospital stay (LOS), specific post-operative complications (wound infections, respiratory complications, intra-abdominal abscess), and anastomotic leak rates
Study design (S)	Randomized controlled trials, prospective comparative studies, and retrospective comparative studies

Search Strategy

A comprehensive literature search was performed in PubMed, Embase, and the Cochrane Library for articles published between January 1, 2000, and December 31, 2024. Boolean operators were used to combine free-text keywords and Medical Subject Headings (MeSH) as follows: (“duodenal ulcer” OR “peptic ulcer”) AND (“perforation” OR “perforated”) AND (“laparoscopic repair” OR “laparoscopy”) AND (“open repair” OR “open surgery”). The Boolean combinations and the number of results retrieved from each database are summarized in Table 2. Reference lists of included articles were also manually screened.

**Table 2:** Boolean search combinations used in each database.

Database	Search terms	Results retrieved
PubMed	(“duodenal ulcer”[MeSH] OR “peptic ulcer”) AND (“perforation” OR “perforated”) AND (“laparoscopic repair” OR “laparoscopy”) AND (“open repair” OR “open surgery”)	146
Embase	(‘duodenal ulcer’/exp OR ‘peptic ulcer’) AND (‘perforation’ OR ‘perforated’) AND (‘laparoscopic repair’ OR ‘laparoscopy’) AND (‘open repair’ OR ‘open surgery’)	128
Cochrane Library	(duodenal ulcer OR peptic ulcer) AND (perforation OR perforated) AND (laparoscopic repair OR laparoscopy) AND (open repair OR open surgery)	38

Study Selection and Data Extraction

Two reviewers independently screened titles, abstracts, and full texts for eligibility. Duplicate records were identified and removed manually by cross-checking database outputs. Disagreements were resolved by discussion and consensus. Data were extracted using a standardized form and included study characteristics, patient demographics, operative approach, conversion rates, operative time, length of hospital stay, overall complications, leak rates, and mortality.

Definitions

For consistency across studies, outcomes were extracted and reported as defined in the original publications. Length of hospital stay (LOS) was reported as mean values in five studies, while one study reported median values. Operative time was reported as mean or mean±standard deviation in most studies, with one study providing median values. Overall complications were reported variably, either as combined morbidity or as specific categories, such as wound infections, respiratory complications, and intra-abdominal abscesses. Conversion was defined as the need to abandon the laparoscopic approach and complete the procedure via laparotomy. Leak rate refers to post-operative duodenal suture leak confirmed clinically and/or radiologically. Mortality was reported as 30-day post-operative or in-hospital death.

Risk of Bias Assessment

Risk of bias was assessed using the Cochrane Risk of Bias-2 (RoB-2) tool for the randomized controlled trial and the Newcastle-Ottawa Scale (NOS) for non-randomized studies [7,8]. Each study was independently assessed by two reviewers, and consensus was reached in cases of disagreement.

Data Synthesis

Due to heterogeneity in study design (randomized and non-randomized), differences in patient selection criteria, and variation in outcome definitions and reporting formats (e.g., length of stay reported as mean versus median, complications defined inconsistently), a quantitative meta-analysis was not feasible. Conducting a pooled analysis under such conditions risked producing misleading or biased results. Therefore, in line with our PROSPERO registration, we performed a structured narrative synthesis of the available evidence. This approach allowed comparison of trends in morbidity, mortality, length of stay, operative time, leak rates, and conversion rates across studies while maintaining methodological transparency.

Results

**Figure 1:**
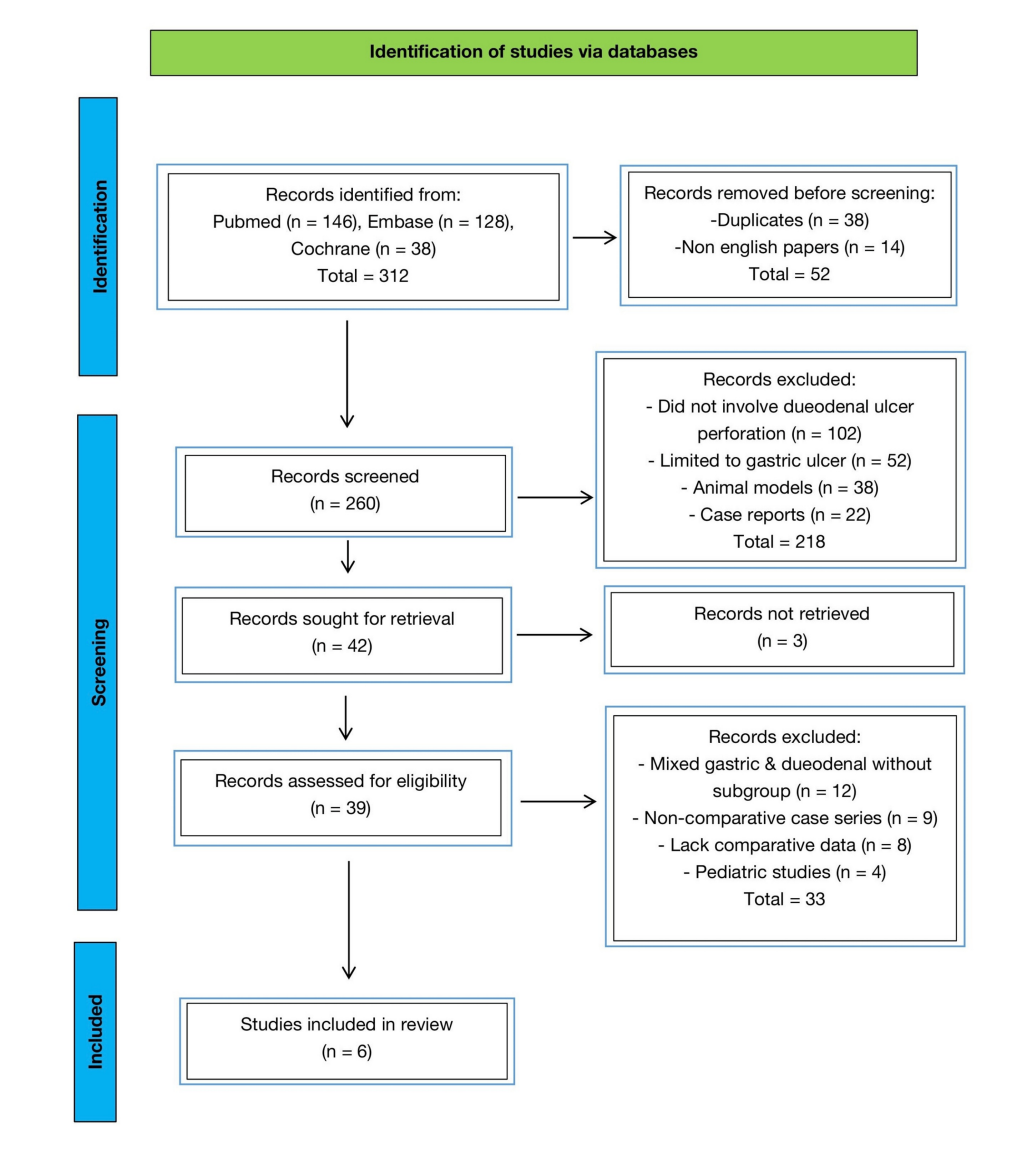
PRISMA 2020 flow diagram of study selection process. PRISMA: Preferred Reporting Items for Systematic Reviews and Meta-Analyses

A total of 312 records were identified through database searches. After removing 52 records (duplicates and non-English studies), 260 records were screened by title and abstract. Of these, 218 were excluded for not meeting the inclusion criteria. The remaining 42 full-text articles were assessed, with 36 excluded for reasons detailed in the PRISMA flow diagram (Figure 1). Six studies met the eligibility criteria and were included in the final review.

The six included studies, summarized in Table 3, were published between 2002 and 2019 and involved a combined total of 739 patients. The number of patients undergoing laparoscopic repair ranged from 14 to 85, while those undergoing open repair ranged from 20 to 174.

**Table 3:** Characteristics of included studies on laparoscopic versus open repair of perforated duodenal ulcer.

Studies	Country	Study design	Lap cases	Open cases	Study period
Mehendale et al. [9]	India	RCT	34	33	2000-2002
Lunevicius and Morkevicius [10]	Lithuania	Retrospective comparative	46	46	1997-2003
Nicolau et al. [11]	Romania	Prospective comparative	85	174	2005-2008
Durai et al. [12]	UK	Retrospective comparative	14	20	2006-2009
Motewar et al. [13]	India	Prospective comparative	70	70	2010-2012
Stepanyan et al. [14]	Armenia	Retrospective comparative	56	61	2014-2017

**Figure 2:**
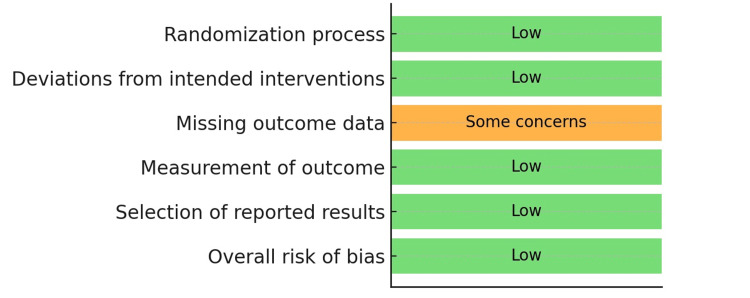
Risk of Bias-2 (RoB-2) traffic light plot. This figure summarizes data reported by Mehendale et al. [9].

One study by Mehendale et al. was a randomized controlled trial, while the remaining five were observational comparative studies (two prospective and three retrospective designs) [9]. Geographically, the studies originated from India, Lithuania, Romania, the United Kingdom, and Armenia. While most studies included all adult patients presenting with perforated duodenal ulcers, Nicolau et al. restricted their analysis to low-risk patients, defined as those aged ≤50 years, with no American Society of Anesthesiologists (ASA) III-IV status, no significant comorbidities, and symptom onset ≤12 hours [11]. Risk of bias assessment showed that the single RCT was judged to have an overall low risk of bias according to the RoB-2 tool, with minor concerns related to missing outcome data (Table 4) [7]. The RoB-2 summary traffic light plot is shown in Figure 2.

**Table 4:** Risk of bias assessment (RoB-2) for the included randomized controlled trial. The table summarizes data reported by Mehendale et al. [9].

Domain	Judgment
Randomization process	Low risk
Deviations from intended interventions	Low risk
Missing outcome data	Some concerns
Measurement of outcome	Low risk
Selection of reported results	Low risk
Overall	Low risk

**Figure 3:**
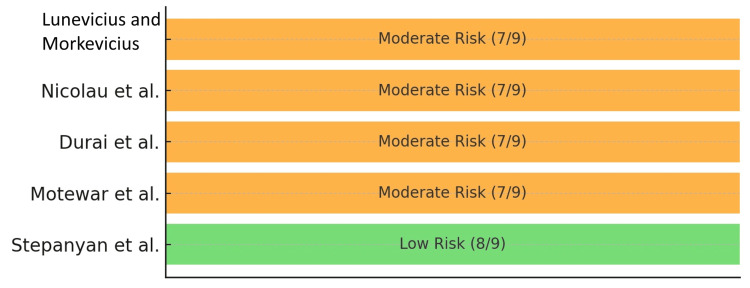
Newcastle-Ottawa Scale traffic light plot.

The Newcastle-Ottawa Scale was used for the remaining non-randomized studies, with scores ranging from 7 to 8 out of 9, indicating moderate to low risk of bias [8]. Stepanyan et al. achieved the highest score, reflecting well-defined inclusion criteria, comparability between groups, and complete follow-up (Table 5) [14]. A visual risk summary for NOS is provided in Figure 3.

**Table 5:** Risk of bias Newcastle-Ottawa Scale for non-randomized studies.

Studies	Selection (0-4)	Comparability (0-2)	Outcome (0-3)	Total (0-9)	Risk level
Lunevicius and Morkevicius [10]	3	1	3	7	Moderate
Nicolau et al. [11]	4	1	2	7	Moderate
Durai et al. [12]	3	1	3	7	Moderate
Motewar et al. [13]	3	1	3	7	Moderate
Stepanyan et al. [14]	4	2	2	8	Low

Across the included studies, the number of laparoscopic and open repairs, as well as the rate of conversion from laparoscopic to open surgery, varied considerably (Table 6). Conversion was most frequent in the trial by Mehendale et al., where 17.8% of laparoscopic cases required open conversion, followed by Motewar et al. with 4.3% and Stepanyan et al. with 2.5%; Durai et al. reported no conversions [9,12-14]. Large perforations, severe peritoneal contamination, and technical difficulties were the main reasons for conversion.

**Table 6:** Conversion rates from laparoscopic to open repair. NA: data not available.

Studies	Lap cases	Open cases	Conversion to open, n (%)
Mehendale et al. [9]	34	33	6 (17.8)
Lunevicius and Morkevicius [10]	46	46	NA
Nicolau et al. [11]	85	174	NA
Durai et al. [12]	14	20	0
Motewar et al. [13]	70	70	3 (4.3)
Stepanyan et al. [14]	56	61	3 (2.5)

Operative time differed between studies (Table 7). Most studies reported mean operative times, with or without standard deviations, while Mehendale et al. presented median values (50 versus 55 minutes). Earlier work, such as Lunevicius and Morkevicius and Nicolau et al., showed longer durations for laparoscopic repair compared with open surgery, whereas more recent studies, notably Stepanyan et al., observed shorter times in the laparoscopic group (94 versus 126 minutes) [9-11,14]. Durai et al. and Motewar et al. reported broadly comparable times between the two approaches [12,13].

**Table 7:** Operative time for laparoscopic and open repair.

Studies	Lap (min)	Open (min)
Mehendale et al. [9] (median)	50	55
Lunevicius and Morkevicius [10] (mean)	76.2	57.3
Nicolau et al. [11] (mean)	85	55
Durai et al. [12] (mean)	67	62
Motewar et al. [13] (mean)	50	48
Stepanyan et al. [14] (mean)	94	126

Length of hospital stay was consistently shorter following laparoscopic repair, reported as mean values in five studies and as median values in one study [9]. Across studies, LOS ranged from 4.0 to 7.8 days for laparoscopic repair compared with 7.8 to 11.7 days for open repair. The most pronounced difference was observed in Stepanyan et al., where the mean stay was 5.0 days for laparoscopic cases and 9.8 days for open cases (Table 8) [14].

**Table 8:** Length of hospital stay following laparoscopic and open repair.

Studies	Lap (days)	Open (days)
Mehendale et al. [9] (median)	4	9
Lunevicius and Morkevicius [10] (mean)	7.8	10.3
Nicolau et al. [11] (mean)	6.1	7.8
Durai et al. [12] (mean)	6.7	8.3
Motewar et al. [13] (mean)	6.5	9.5
Stepanyan et al. [14] (mean)	5.0	9.8

Anastomotic leak rates were low in both groups, typically between 0% and 7% for laparoscopic repair and 1.4% to 4.9% for open repair (Table 9). No study reported a statistically significant difference in leak incidence between the two techniques.

**Table 9:** Anastomotic leak rates in laparoscopic and open repair. NA: data not available; n: number of patients with the outcome; N: total number of patients in that group

Studies	Lap leak, n/N (%)	Open leak, n/N (%)
Mehendale et al. [9]	0/34	NA
Lunevicius and Morkevicius [10]	3/46 (7)	1/46 (2)
Nicolau et al. [11]	2/85 (2.4)	NA
Durai et al. [12]	NA	NA
Motewar et al. [13]	2/70 (2.9)	1/70 (1.4)
Stepanyan et al. [14]	3/56 (5.4)	3/61 (4.9)

Mortality rates were generally lower with laparoscopy (0-4.3%) compared to open repair (0-27.9%) (Table 10). The largest difference was reported by Stepanyan et al. (1.8% versus 27.9%), while two studies found no deaths in either group [14].

**Table 10:** Mortality rates in laparoscopic and open repair. NA: data not available; n: number of patients with the outcome; N: total number of patients in that group

Studies	Lap mortality, n/N (%)	Open mortality, n/N (%)
Mehendale et al. [9]	NA	NA
Lunevicius and Morkevicius [10]	0/46	4/46 (9)
Nicolau et al. [11]	0/85	0/174
Durai et al. [12]	0/14	1/20 (5)
Motewar et al. [13]	3/70 (4.3)	6/70 (8.6)
Stepanyan et al. [14]	1/56 (1.8)	17/61 (27.9)

Overall complication rates were also lower with laparoscopic repair in most studies (Table 11). Stepanyan et al. reported complications in 10.7% of laparoscopic cases compared to 44.3% of open cases, and Lunevicius and Morkevicius reported 13% versus 25% cases, respectively [10,14]. Nicolau et al., limited to low-risk patients, observed a smaller difference (5.8% versus 8.6%), while Durai et al. and Motewar et al. provided detailed breakdowns of specific complications, such as wound infections, respiratory issues, and intra-abdominal abscesses [11-13].

**Table 11:** Overall post-operative complication rates. NA: data not available; n: number of patients with the outcome; N: total number of patients in that group

Studies	Lap complications, n/N (%)	Open complications, n/N (%)
Mehendale et al. [9]	NA	NA
Lunevicius and Morkevicius [10]	6/46 (13)	12/46 (25)
Nicolau et al. [11]	5/85 (5.8)	15/174 (8.6)
Durai et al. [12]	Wound: 1; chest: 1	Wound: 4; chest: 3
Motewar et al. [13]	Resp: 3; abscess: 0; wound: 3; adhesion: 0	Resp: 14; abscess: 4; wound: 18; adhesion: 15
Stepanyan et al. [14]	6/56 (10.7)	27/61 (44.3)

**Figure 4:**
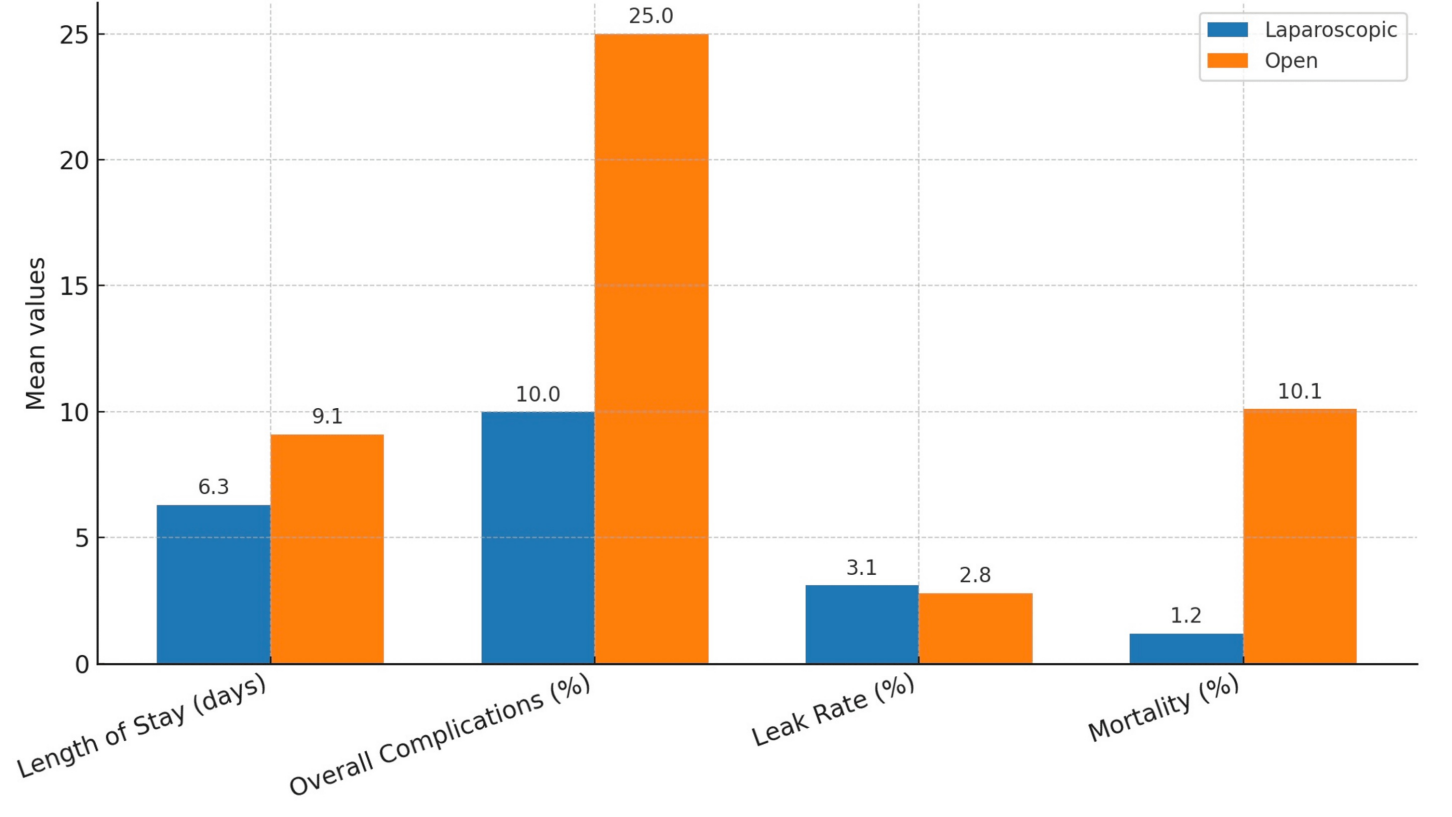
Comparative bar chart showing the average length of stay, complication rate, leak rate, and mortality for laparoscopic and open repair of perforated duodenal ulcer across included studies. This figure summarizes data reported by Mehendale et al., Lunevicius and Morkevicius, Nicolau et al., Durai et al., Motewar et al., Stepanyan et al. [9-14]. This figure represents a descriptive summary of reported outcomes. No formal meta-analysis or statistical pooling was performed due to heterogeneity in study design and reporting formats. LOS: length of stay

To support visual interpretation of the findings, a descriptive bar chart was created to compare the reported average values of key outcomes, including length of stay, complication rate, leak rate, and mortality, between laparoscopic and open repair across the included studies (Figure 4). This figure illustrates the general trend toward improved outcomes with laparoscopic repair, consistent with the tabulated data.

Discussion

This systematic review focused specifically on adults with perforated duodenal ulcers and compared post-operative outcomes following laparoscopic versus open repair. In line with the review’s primary objectives, the synthesis shows that laparoscopy is associated with lower overall morbidity and mortality compared with open repair, while leak rates are comparable across approaches. Secondary outcomes also generally favor laparoscopy; shorter length of stay (LOS) was consistently reported, and operative time initially longer in early series was broadly similar or shorter in more recent work, reflecting a learning-curve effect. Collectively, these findings support the selective use of laparoscopy in stable patients while acknowledging that open repair remains necessary in specific clinical contexts.

All six studies reported shorter hospital stays after laparoscopy, with differences ranging from modest to clinically meaningful. This finding aligns with the established benefits of minimally invasive surgery -reduced pain, earlier mobilization, and faster return of bowel function - which likely contribute to earlier discharge. The most pronounced LOS benefit in our cohort was reported by Stepanyan et al., while other studies showed more modest but consistent gains [14]. These results are directionally concordant with broader literature on peptic ulcer perforation (not limited to the duodenum), including the contemporary meta-analysis by Chan et al., which also found shorter LOS and fewer wound-related complications with laparoscopy [15]. Although one included trial reported LOS as medians rather than means, the overall pattern remains uniform across the evidence base [9].

The overall complication rate typically favored laparoscopy. In large part, this appears driven by lower wound infection and respiratory complications, with plausible mechanistic advantages of smaller incisions and decreased post-operative pain. Stepanyan et al. reported the largest absolute difference, whereas studies with lower-risk profiles (e.g., Nicolau et al.) showed smaller absolute gaps, consistent with baseline risk influencing observed effect size [11,14]. Importantly, anastomotic (suture) leak rates were low and comparable between groups across the series, suggesting that, with appropriate patient selection and technique, laparoscopic closure does not compromise repair integrity.

Mortality outcomes tended to favor laparoscopic repair, with the most substantial difference again observed in Stepanyan et al., while other cohorts reported either lower mortality with laparoscopy or no deaths in either arm [14]. These patterns likely reflect a combination of true peri-operative benefit (less surgical trauma, fewer pulmonary complications) and case selection, because surgeons may preferentially choose laparoscopy for hemodynamically stable patients with more favorable intra-abdominal conditions. This interpretation is consistent with reasons for conversion cited across studies (large perforations, severe peritoneal contamination, technical difficulty), which indicate that the sickest or most complex presentations often necessitate or revert to open surgery.

Operative duration showed temporal heterogeneity. Earlier series (e.g., studies by Lunevicius and Morkevicius, Nicolau et al.) tended to report longer laparoscopic times, likely reflecting the learning curve for intracorporeal suturing, contamination management, and limited early access to advanced energy devices [10,11]. Later work (e.g., study by Stepanyan et al.) demonstrates shorter laparoscopic times, while others reported broadly comparable durations between approaches (e.g., Durai et al., Motewar et al., and the median-based report in Mehendale et al.) [9,12-14]. This evolution underscores how surgeon experience, institutional volume, and technology adoption can modulate time-related outcomes without negating the recovery advantages that laparoscopy delivers.

Taken together, the data suggest that laparoscopy should be considered the preferred approach for stable adult patients with perforated duodenal ulcers when appropriate expertise and resources are available. Conversely, open repair remains indispensable for patients with hemodynamic instability, gross contamination, large or friable perforations, or where laparoscopic expertise and equipment are limited. This pragmatic, context-sensitive strategy mirrors conversion triggers observed across the included studies and aligns with real-world surgical decision-making.

The strength of this review lies in its focused anatomical scope (duodenum only), explicit comparative design, and adherence to PRISMA 2020 with a pre-registered protocol [6]. Nevertheless, several limitations temper inference. First, the evidence base is small (six studies, 739 patients) and heterogeneous in design (one RCT and five observational studies) and reporting [9]. Core outcomes (LOS, operative time) were presented as means in most studies but as medians in one study, and complication definitions varied (composite versus category-specific counts), limiting direct comparability and precluding a valid pooled estimate. Second, selection bias is likely, as laparoscopy was often performed in more stable patients, potentially inflating the observed benefits on mortality and complications. Third, reporting granularity was variable; some studies lacked measures of dispersion or did not provide standardized 30-day endpoints, and one study restricted inclusion to low-risk cohorts, thereby limiting generalizability [11]. Finally, by design, we excluded non-English literature and grey literature, prioritizing methodological completeness and outcome detail over maximal breadth, which may introduce language or publication bias; however, this choice maintains clarity and replicability of the extracted outcomes.

To resolve residual uncertainty and support guideline-level recommendations, future research should prioritize multicentre randomized trials with standardized outcome definitions (e.g., 30-day morbidity and mortality, validated complication grading), uniform time metrics (means with SD or medians with IQR, pre-specified), and clear selection criteria for laparoscopic versus open approaches (including hemodynamic status, contamination grade, perforation size/location). Prospective registries could capture real-world conversion thresholds, learning-curve effects, and resource utilization (LOS, readmissions), while economic evaluations should quantify cost-effectiveness across diverse settings. Finally, harmonized reporting would enable robust meta-analysis in the future and more precise patient-level recommendations.

## Conclusions

This systematic review demonstrates that laparoscopic repair of perforated duodenal ulcer offers clear advantages over open repair in appropriately selected patients. Across the available comparative studies, laparoscopy was associated with shorter hospital stays, fewer complications, and lower mortality rates, while leak rates were comparable between the techniques. Although earlier studies reported longer operative times, more recent evidence shows that this difference diminishes with increasing surgeon experience and advances in laparoscopic technology. Open repair remains indispensable for patients with hemodynamic instability, severe contamination, or extensive perforations where laparoscopy is not feasible. Further multicentre randomized trials with standardized outcome reporting are required to confirm these findings, define optimal patient selection, and evaluate cost-effectiveness across diverse healthcare settings.
